# Improved Effect of Spinach Extract on Physical Performance: A Systematic Review of Randomized Controlled Trials

**DOI:** 10.7759/cureus.77840

**Published:** 2025-01-22

**Authors:** Jimmy Wen, Burhaan Syed, Ihab Abed, Dave Manguerra, Mouhamad Shehabat, Daniel I Razick, Denise Nadora, Dawnica Nadora, Muzammil Akhtar, David Pai

**Affiliations:** 1 Physical Medicine and Rehabilitation, California Northstate University College of Medicine, Elk Grove, USA; 2 Surgery, California Northstate University College of Medicine, Elk Grove, USA; 3 Internal Medicine, California Northstate University College of Medicine, Elk Grove, USA; 4 Dermatology, California Northstate University College of Medicine, Elk Grove, USA; 5 Nephrology, California Northstate University College of Medicine, Elk Grove, USA

**Keywords:** nitrates, spinach, spinach extract, sports performance, supplements

## Abstract

Nitric oxide is a key regulator of many systems in the human body and has become a popular supplement for sports, given its potential to increase health and physical performance. Spinach extract contains a rich amount of nitrates, and we aim to examine the effects of its supplementation on physical performance, body composition, and safety profile. A systematic review following the guidelines from the Preferred Reporting Items for Systematic Reviews and Meta-Analyses (PRISMA) was performed in PubMed, Embase, and Cochrane Library for randomized controlled trials (RCTs) reporting on physical performance parameters after spinach extract supplementation. Study variables extracted included author, publication date, study year, number of patients, male/female ratio, mean age, mean follow-up, dose regimen, pre- and post-intervention patient-reported outcomes, and complications. Four RCTs (three red spinach, one green spinach), with 94 patients (45.7% male, 54.3% female), mean age of 29.4 years (20.5 to 58.9) and mean follow-up time of 43.8 days (7 to 84). Dosages ranged from 1 to 2 g. Spinach extract demonstrated significant improvements in most physical performance parameters, mixed results on body composition, and no complications were reported. Spinach extract demonstrates promising improvements in physical performance and safety profile as an ergogenic aid. However, more research is required to determine optimal dosing regimens and their effects in different patient populations.

## Introduction and background

Nitric oxide (NO-) is an important regulatory molecule that functions in many systems in the human body. It plays key roles as a vasodilator, anticoagulant, skeletal muscle modulator, anti-oxidant, and insulin modulator [1]. NO- has a short half-life as it can be easily oxidized. Thus, it travels in more stable forms in the body, such as nitrate (NO3-) and nitrite (NO2-) [1-2]. NO3- can be converted to NO- through the NO3-NO2-NO- pathway or converted to NO2- in the mouth or gastrointestinal (GI) tract by commensal bacteria [2].

Endogenously, the NO- synthase (NOS) dependent pathway synthesizes NO- from L-arginine. The NO- can then be used directly or oxidized into NO3- or NO2- [3]. In endothelial cells, calcium signaling of endothelial NOS (eNOS) can lead to NO- generation from L-arginine. This eventually leads to downstream interactions with cGMP and myosin light chain to modulate vascular smooth muscle tone [3]. In cardiac myocytes, different NOS isoforms have similarly been shown to be important for normal function and remodeling [4]. Nuclear signaling and transcription of different NOS isoforms is the primary method by which cells modify the function of NO- [4]. Additionally, it has been shown that certain behavior modifications, such as regular exercise activity, increase the body's innate NO- generation by upregulating eNOS expression, thus increasing the blood's basal levels of NO3- [5]. Furthermore, NO- plays an important role in vasodilation during states of ischemic hypoxia, where perfusion may be preserved [2].

The International Olympic Committee has designated five supplements to enhance physical performance: caffeine, beta-alanine, sodium bicarbonate, beetroot juice, and creatine. Beetroot juice and NO3- are the only two of the five that come from a natural source [6]. There are many sources of dietary NO3-, notably leafy greens such as spinach, beets, Chinese flat cabbage, and arugula [7]. However, spinach extract (red/amaranth and green/spinacia) contains a rich amount of NO3- and several other bioactive compounds such as carotenoids, calcium, anti-oxidants, and polyphenols that may offer additional benefits. Green spinach also contains a high ecdysteroid content, demonstrating anabolic strength and muscle mass benefits.

NO3- has become a popular supplement for sports, given its potential to increase health and physical performance. However, the literature shows mixed results, with some studies showing improved cardiovascular endurance via increased time-to-exhaustion and improved aerobic performance at varying distances [8-9]. It also decreases the oxygen cost of submaximal intensity exercises in inactive and recreational men and women [10]. This systematic review aims to compile and evaluate the effects of spinach extract supplementation on physical performance, body composition, and safety profile. We hypothesize that spinach extract will positively affect physical performance with an overall low rate of complications.

## Review

Methods

Search Strategy

This systematic review followed the guidelines established by the Preferred Reporting Items for Systematic Reviews and Meta-Analyses (PRISMA). A search was performed in PubMed, Embase, and Cochrane Library on May 6, 2024. The query of articles was performed using the following search phrases: “spinach extract”, “muscle”, “performance”, “outcomes”, “longevity”, “efficacy”, “athlete”, and “aerobic”. No restrictions were set on the search strategies.

Article Selection

A PICOT (patient, intervention, comparison, outcome, time) method was used to determine our search strategy. The patient population was defined as individuals of all age ranges taking spinach extract. The intervention included the consumption of spinach extract alone or compared to a placebo or an active comparator. Studies were included if they reported any physical performance outcomes post-supplementation of spinach extract compared to placebos. There were no restrictions on dosage, duration of use, or outcome measures. The outcomes in this study consisted of physical performance parameters, body composition, rates of complications, and patient-reported outcomes (PROs). Studies with at least one-week follow-up were included to examine the effects outside of acute supplementation. Exclusion criteria included case reports, review articles, animal studies, conference abstracts, articles not in English, and papers reporting no outcomes or outcomes other than those specified in the inclusion criteria. This review was registered in the PROSPERO database as CRD42024550994.

Two reviewers independently reviewed all studies using predetermined eligibility criteria during the title/abstract and full-text screening. A third reviewer was consulted if the initial two reviewers did not agree on whether to include or exclude a study. Additionally, all included articles underwent a thorough reference search to identify any additional studies that could be added to the systematic review.

Study Quality

The Cochrane Collaboration's Risk of Bias (RoB) tool was utilized to evaluate the quality and RoB of the included articles [11]. Two reviewers assessed each article based on several criteria, including random sequence generation, allocation concealment, blinding of participants and personnel, blinding of outcome assessment, incomplete outcome data, selective outcome reporting, and other sources of bias. Any disagreements were resolved through thorough re-reviewing until a consensus was reached.

The following information was gathered from each study: study details (author, title, publication year, year of study), patient demographics (number of patients, male/female ratio, average age, average follow-up), dosage of spinach extract, pre- and post-intervention PROs), and any complications. All the collected data was organized in Google Sheets (Google LLC, Mountain View, CA, USA) for analysis, and tables were created using Microsoft Word (Microsoft Corporation, Redmond, WA, USA) to present the data visually. Descriptive statistics (such as means, percentages, and standard deviations) are included in this review when applicable and available. Although a meta-analysis was planned to analyze combined pre- to post-intervention PROs, it could not be performed due to the heterogeneity of the included studies.

Results

**Figure 1:**
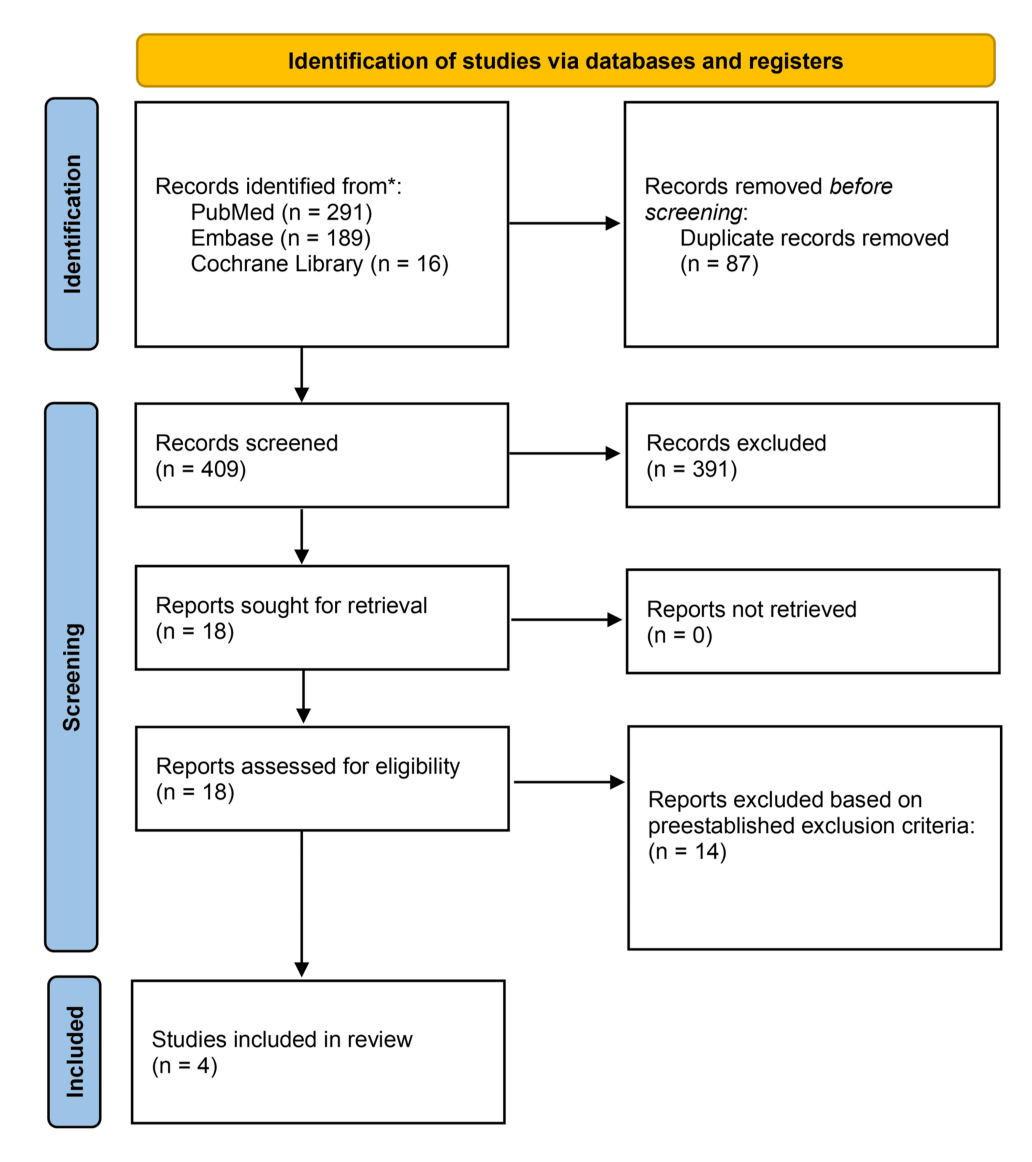
Preferred Reporting Items for Systematic Reviews and Meta-Analyses (PRISMA) diagram of included studies

The initial search resulted in 409 articles for screening. With the removal of 87 duplicates, 391 studies were excluded in the title and abstract screening. After screening 18 full texts, four studies remained to be included in this systematic review, as shown in Figure 1.

Demographic and Study Characteristics

In the four studies reviewed, there were 94 patients, with 45.7% male and 54.3% female [12-15]. The average age of the patients was 29.4 years (ranging from 20.5 to 58.9), and the average follow-up time was 43.8 days (ranging from 7 to 84). Two studies included only male participants [12-13]. The average NO3- content in the red spinach extract (RSE) was 6.9% (ranging from 0.71% to 9%). The complete demographic data can be found in Table 1.

**Table 1:** Patient demographics and study characteristics NO3-: nitrate, LOE: level of evidence, NR: not reported, M: male, F: female

Author	Journal	Study year	LOE	Number of patients (M/F)	Age (years)	Follow-up (weeks)	NO3- content (% of total spinach extract dosage)	Composition of spinach extract
Pérez-Piñero et al., 2021 [15]	Nutrients	September 2020-June 2021	2	8/37	58.9 ± 6.1	1	14.18 mg (0.71%)	Per 100 g of extract: carbohydrates 29.3 g, protein 20.4 g, fat <0.5 g, dietary fiber 9.6 g, sodium 10.3 g NaCl, vitamin C <15 mg, calcium39.5 mg, iron 34.3 mg, potassium 11.4 mg, and total sugars 20.7 mg
Haynes et al., 2021 [12]	Sports	September 2019-March 30, 2020	2	10/0	22.6 ± 3.2	1	180 mg (9%)	NR
Townsend et al., 2022 [13]	Journal of Dietary Supplements	NR	2	16/0	20.5 ± 1.7	11	180 mg (9%)	NR
Gonzalez et al., 2021 [14]	Journal Strength Conditioning Research	NR	2	9/8	M: 22.2 ± 3.8 y	12	90 mg (9%)	Citric acid, natural flavor, malic acid, *Stevia rebaudiana* leaf extract, beet juice color, and calcium silicate
					F: 22.8 ± 3.5 y			

The four studies assessed in this review demonstrated a low RoB for most domains. Pérez-Piñero et al. had a potential RoB for domains one, three, and four and a high RoB for seven [15]. Haynes et al. had a low RoB for all domains except for an unclear potential for bias in domain three [12]. Townsend et al. had a potential bias in domains three and four and a high RoB in domain seven [13]. Lastly, Gonzalez et al. showed a high RoB in domains one, two, three, and seven, with potential bias in domain four [14]. The Cochrane RoB breakdown can be found in Table 2.

**Table 2:** Cochrane Risk of Bias (RoB)

Author	Sequence generation	Allocation concealment	Blinding of participants and personnel	Blinding of outcome assessors	Incomplete outcome data	Selective outcome reporting	Other sources of bias
Pérez-Piñero et al., 2021 [15]	Unsure	Low	Unsure	Unsure	Low	Low	High
Haynes et al., 2021 [12]	Low	Low	Unsure	Low	Low	Low	Low
Townsend et al., 2022 [13]	Low	Low	Unsure	Unsure	Low	Low	High
Gonzalez et al., 2021 [14]	High	High	High	Unsure	Low	Low	High

Physical Performance

All four studies were double-blinded, randomized controlled trials. There were no common performance parameters between any of the studies. A complete summary of all physical parameters for each study can be found in Table 3.

**Table 3:** Effect of spinach extract on physical performance outcomes NM: Newton meter, RM: repetition maximum, ∆%SmO2: loss of muscle oxygenation, SmO2RecT: muscle reoxygenation time, SmO2RecSlope: muscle oxygen resaturation rate, SmO2Peak: highest SmO2 value achieved in a rest period, AU: arbitrary units, PRE: immediately prior to exercise, IP: immediately post-exercise, NR: not reported * between baseline and end of the study for the intervention group, ** between baseline and end of the study for the placebo group, *** between the intervention and placebo group

Author	Intervention dose	Variable	Placebo pre-intervention	Pre-intervention for the experimental group	Placebo post-intervention	Post-intervention	p-value
Pérez-Piñero et al., 2021 [15]	0 vs 2 g / day	60° s^-1 ^peak torque Nm	93.6 ± 16.9	91.6 ± 28.1	101.4 ± 18.0	107.2 ± 26.6	0.001*
							0.001**
							0.007***
		60° s^-1 ^Total work for 1RM, J	85.2 ± 13.6	83.3 ± 23.3	94.3 ± 20.0	101.5 ± 24.7	0.001*
							0.001**
							0.009***
		60° s^-1 ^total work, J	384.7 ± 85.6	390.3 ± 115.3	445.6 ± 92.4	474.4 ± 118.3	0.001*
							0.001**
							0.289***
		60° s^-1 ^average power, W	54.2 ± 9.8	53.6 ± 17.8	60.3 ± 13.2	63.4 ± 17.2	0.001*
							0.001**
							0.109***
		180° s^-1 ^peak torque, Nm	60.9 ± 12.2	58.6 ± 19.5	64.2 ± 12.9	72.4 ± 19.3	0.001*
							0.001**
							0.002***
		180° s^-1 ^total work for 1RM, J	62.5 ± 12.3	62.2 ± 22.3	68.4 ± 14.3	79.2 ± 23.5	0.001*
							0.001**
							0.005***
		180° s^-1 ^total work, J	287.7 ± 61.0	285.3 ± 100.9	317.3 ± 66.8	363.3 ± 104.5	0.001*
							0.001**
							0.007***
		180° s^-1 ^average power, W	94.4 ± 22.3	93.3 ± 35.6	102.2 ± 24.6	115.1 ± 31.9	0.001*
							0.001**
							0.027***
		90° s^-1 ^peak torque, Nm	119.7 ± 34.7	124.7 ± 47.3	126.9 ± 28.9	143.2 ± 45.1	0.001*
							0.001**
							0.005***
		90° s^-1 ^average peak torque, Nm	114.4 ± 34.2	118.7 ± 44.5	121.0 ± 29.4	136.4 ± 44.7	0.001*
							0.001**
							0.002***
		Handgrip strength right hand, kg	24.8 ± 8.7	25.7 ± 8.2	24.9 ± 7.9	26.4 ± 7.1	0.001*
							0.001**
							0.449***
		Handgrip strength left hand, kg	23.3 ± 7.8	23.3 ± 7.4	24.3 ± 6.9	25.4 ± 7.4	0.001*
							0.001**
							0.247***
		Maximal dynamic force (1RM), kg	41.4 ± 14.0	43.2 ± 13.8	57.3 ± 15.1	60.1 ± 16.7	0.001*
							0.001**
							0.729***
Haynes et al., 2021 [12]	0 vs 2 g/ day	∆%SmO_2_ (set 1)	NR		69.0 ± 12.6	63.2 ± 19.7	N.S**
		SmO_2_RecT (s)			57.8 ± 9.3	60.8 ± 27.3	N.S**
		SmO_2_RecSlope			1.26 ± 0.44	1.14 ± 0.33	N.S**
		SmO_2_Peak (%)			87.0 ± 3.24	85.3 ± 3.43	N.S**
		∆%SmO_2_ (set 2)			61.4 ± 15.8	69.4 ± 15.8	N.S**
		SmO_2_RecT (s) (set 2)			60.2 ± 14.0	55.6 ± 18.8	N.S**
		SmO_2_RecSlope (set 2)			1.07 ± 0.32	1.17 ± 0.41	N.S**
		SmO_2_Peak (%) (set 2)			87.3 ± 3.57	86.1 ± 4.89	N.S**
		∆%SmO_2_ (set 3)			60.7 ± 12.4	59.7 ± 16.1	N.S**
		SmO_2_RecT (s) (set 3)			58.7 ± 9.7	53.2 ± 58.7	N.S**
		SmO_2_RecSlope (set 3)			0.86 ± 0.80	0.91 ± 0.56	N.S**
		SmO_2_Peak (%) (set 3)			86.8 ± 3.60	84.1 ± 5.28	N.S**
		∆%SmO_2_ (set 4)			61.3 ± 17.1	61.5 ± 5.3	N.S**
		SmO_2_RecT (s) (set 4)			60.9 ± 12.1	52.6 ± 17.4	N.S**
		SmO_2_RecSlope (set 4)			0.92 ± 0.49	1.06 ± 0.68	N.S**
		SmO_2_Peak (%) (set 4)			86.1 ± 4.76	84.6 ± 6.38	N.S**
		∆%SmO_2_ (set 5)			66.5 ± 12.3	62.0 ± 21.3	N.S**
		Focus (cm)	7.6 ± 2.0	8.3 ± 2.5	PRE: 8.7 ± 2.7	PRE: 9.0 ± 2.7	<0.05*
					IP: 9.9 ± 1.8	IP: 9.8 ± 2.8	<0.05**
		Energy (cm)	7.2 ± 2.9	7.7 ± 2.7	PRE: 8.5 ± 2.6	PRE: 8.8 ± 2.3	N.S
					IP: 8.1 ± 2.7	IP: 7.4 ± 2.5	
		Fatigue (cm)	4.4 ± 2.9	4.7 ± 3.2	PRE: 4.5 ± 2.3	PRE: 5.0 ± 2.7	<0.05*
					IP: 8.9 ± 3.2	IP: 10.1 ± 2.4	<0.05**
		Muscle pump (cm)	4.3 ± 4.1	3.9 ± 3.1	PRE: 6.6 ± 3.1	PRE: 6.2 ± 3.3	<0.05*
					IP: 11.0 ± 2.4	IP: 11.2 ± 2.2	<0.05**
		Rating of perceived exertion (AU)	NR		IP: 9.9 ± 1.8	IP: 8.3 ± 0.7	N.S
		Congruence (ms)	751.2 ± 148.0	720.1 ± 129.4	PRE: 648.6 ± 96.7	PRE: 625.0 ± 72.9	<0.05*
					IP: 614.9 ± 90.8	IP: 607.3 ± 83.1	<0.05**
		Incongruence (ms)	877.4 ± 105.5	831.9 ± 106.1	PRE: 754.8 ± 91.3	PRE: 749.0 ± 92.2	<0.05*
					IP: 772.5 ± 111.0	IP: 740.9 ± 113.4	<0.05**
								Stroop effect (ms)	113.6 ± 72.4	111.8 ± 107.1	PRE: 106.1 ± 95.4	PRE: 126.0 ± 64.5	N.S
		IP: 132.0 ± 54.5	IP: 135.6 ± 113.8										
		Townsend et al., 2022 [13]	0 vs 2 g/ day	Bench press 1RM (kg)	100.6 ± 21.1	107.7 ± 18.2	123.5 ± 20.8	121.3 ± 21.6	<0.001*
0.298***								
Peak power (W)	1130.9 ± 137.1			1024.4 ± 194.6	1108.1 ± 143.0	1134.0 ± 115.4	0.189*
							0.095***
Relative peak power (W/kg)	12.5 ± 1.84			11.4 ± 1.42	12.6 ± 1.71	12.8 ± 1.86	0.214*
							0.128***
Mean power (W)	682.3 ± 108.0			651.1 ± 80.9	682.4 ± 86.9	699.3 ± 43.8	0.133*
							0.135***
Relative mean power (W/kg)	7.63 ± 1.40			7.33 ± 0.87	7.73 ± 1.03	7.94 ± 1.19	0.195*
							0.178***
Minimum power (W)	409.9 ± 105.7			411.0 ± 50.0	413.7 ± 91.8	427.6 ± 32.5	0.254*
							0.978***
Relative minimum power (W/kg)	4.52 ± 1.23			4.69 ± 1.00	4.69 ± 1.10	4.87 ± 0.87	0.289*
							0.970***
Fatigue index (%)	59.0 ± 6.8			53.6 ± 11.3	57.9 ± 5.7	55.9 ± 7.3	0.981*
							0.345***
Gonzalez et al., 2021 [14]	0 vs 1 g/ day	4-km time (s)	NR		410.6±31.3	404.6 ± 24.3	0.013***
		Average power (W)			181.6±35.1	185.9±32.2	0.022***
		Relative power (W/kg)			2.46±0.40	2.53±0.44	0.009***
		Cadence (rpm)			76.0±7.0	76.1±5.2	0.884***
		Average speed (km/h)			35.3±2.5	35.7±2.2	0.010***
		Muscle fatigue (au)			9.7±2.4	10.8±2.0	0.187***
		RPE (au)			7.1±1.2	7.2±1.3	0.948***

In a study conducted by Gonzalez et al., the effects of RSE on cycle time trial performance were investigated. Physically active men and women were given either 1 g of RSE or a placebo over seven days. The results showed a significant difference between the RSE and placebo groups in terms of the 4-km time (p=0.013), average power (p=0.022), relative power (p=0.009), and average speed (p=0.01). Additionally, differences between men and women for these parameters were also noted (p<0.022) [14].

In a study by Haynes et al., 10 males received either a placebo (maltodextrin) or 2 g of RSE over seven days [12]. The study found no significant differences between the RSE and placebo groups for any muscle oxygenation parameter (loss of muscle oxygenation, muscle reoxygenation time, muscle oxygen resaturation rate, and peak muscle oxygenation during the bench press). However, both groups showed a significant increase in focus and muscle pump during bench press from baseline to immediately before exercise (p<0.05). Both groups also significantly increased from baseline to after exercise for focus, energy, fatigue, and muscle pump (p<0.05). Regarding the Stroop test, there was a significant decrease in the congruence (matching the color with its word) and incongruence (matching the color with the word in a different colored font) time before and after exercise for both groups. Additionally, the RSE and placebo groups had a significantly lower incongruence time after exercise compared to before exercise.

Townsend et al. explored RSE and placebo effects in male collegiate baseball athletes over 11 weeks [13]. Besides a significant increase from baseline for one repetition maximum bench press for both groups, no other parameter had a significant difference (peak and relative peak power (W, W/kg), mean and relative mean power (W, W/kg), minimum and relative minimum power (W, W/kg), and fatigue index during the Wingate anaerobic test), either from baseline or between the two groups.

Finally, in Pérez-Piñero et al., an older population of adults above the age of 50 was given either a placebo (maltodextrin) or 2 g of green spinach extract (GSE) a day for 12 weeks [15]. For the experimental group, it was found that there was a significant increase from the baseline measurements for all parameters (p=0.001, p=0.004 for left-hand grip strength) except for right-hand grip strength. The placebo group showed a notable increase from baseline for maximal dynamic force, muscle function at 60° s-1 and 90° s-1, and total work for one and five repetitions at 180° s-1 (p<0.05). Statistically significant differences were observed between the RSE and placebo groups for all parameters except for total work and average power at 60° s-1, handgrip strength, and maximal dynamic force (p<0.05).

Body Composition

In the three studies that specifically examined RSE, heart rate, systolic blood pressure, and diastolic blood pressure were common body parameters measured. However, two studies did not report exact values [12-13]. Two studies found a significant change in heart rate from baseline to the end of the experiment for both groups (p<0.001) [12,14]. Gonzalez et al. also observed a significant decrease in diastolic blood pressure in the RSE group compared to the placebo group (p=0.017). In Pérez-Piñero et al., measurements such as body weight (kg), fat, lean, and appendicular skeletal muscle mass (kg) were recorded with GSE [15]. There was a significant increase from baseline in the RSE group for fat, lean, and appendicular skeletal muscle mass (p<0.05) and for lean and muscle mass in the placebo group (p<0.05). However, there were no significant differences between the two groups. Additional body composition parameters can be found summarized in Table 4.

**Table 4:** Effect of spinach extract on body composition and vital signs RM: repetition maximum, PRE: immediately prior to exercise, IP: immediately post-exercise, NR: not reported * between baseline and end of the study for the intervention group, ** between baseline and end of the study for the placebo group, *** between intervention and placebo group

Author	Intervention dose	Variable	Placebo pre-intervention	Pre-intervention for the experimental group	Placebo post-intervention	Post-intervention	p-value
Pérez-Piñero et al., 2021 [15]	0 vs 2 g/ day	Body weight, kg	68.0 ± 9.7	69.3 ± 8.2	67.7 ± 9.7	69.0 ± 8.4	0.374*
							0.361**
							0.975***
		Fat mass, kg	28.3 ± 4.9	27.8 ± 4.1	27.7 ± 4.8	27.4 ± 4.3	0.05*
							0.014**
							0.615***
		Lean mass, kg	37.7 ± 7.4	39.0 ± 6.6	38.1 ± 7.3	39.6 ± 6.8	0.001*
							0.02**
							0.254***
		Muscle mass, kg	37.5 ± 7.5	38.8 ± 6.9	37.9 ± 7.4	39.4 ± 6.9	0.001*
							0.16**
							0.429***
		Appendicular skeletal muscle mass, dominant leg, kg	6.0 ± 1.4	6.2 ± 1.3	6.0 ± 1.3	6.3 ± 1.3	0.013*
							0.143**
							0.463***
Haynes et al., 2021 [12]	0 vs 2 g/ day	Heart rate	NR				<0.001*
							0.301***
		Systolic blood pressure					0.260*
							0.717***
		Diastolic blood pressure					0.948*
							0.216***
Townsend et al., 2022 [13]	0 vs 2 g/ day	Body mass (kg)	91.2 ± 9.0	89.7 ± 13.7	90.6 ± 7.4	89.6 ± 13.2	0.646*
							0.625***
		Fat free mass (kg)	75.2 ± 5.0	76.3 ± 7.8	77.4 ± 4.9	77.8 ± 8.0	<0.001*
							0.342***
		Fat mass (kg)	15.9 ± 9.2	13.5 ± 7.1	13.3 ± 7.1	11.8 ± 5.9	0.005*
							0.428***
		Body fat (%)	16.9 ± 8.7	14.4 ± 5.6	14.3 ± 6.9	12.7 ± 4.8	0.002*
							0.450***
		Rectus femoris muscle thickness (cm)	2.53 ± 0.34	2.59 ± 0.36	2.82 ± 0.31	2.88 ± 0.36	<0.001*
							0.920***
		Vastus lateralis muscle thickness (cm)	1.72 ± 0.24	1.76 ± 0.19	1.86 ± 0.24	1.94 ± 0.29	<0.001*
							0.620***
		Heart rate	NR				0.905*
							0.374***
		Systolic blood pressure					0.044*
							0.563***
		Diastolic blood pressure					0.086*
							0.490***
Gonzalez et al., 2021 [14]	0 vs 1g / day	Heart rate	69.2 ± 11.2	67.6 ± 8.9	PRE: 65.6 ± 8.4	PRE: 66.2 ± 8.6	IP: <0.001*
					IP: 149.5 ± 13.4	IP:149.9 ± 13.4	
		Systolic blood pressure	117.0 ± 8.0	119.2 ± 5.1	PRE: 118.3 ± 8.8	PRE:118.8 ± 7.0	IP: <0.001*
					IP: 149.2 ± 8.2	IP: 149.3 ± 11.1	
		Diastolic blood pressure	75.1 ± 6.7	73.8 ± 5.2	PRE:75.2 ± 7.0	PRE:76.4 ± 4.3	IP: <0.05***
					IP:70.1 ± 5.0	IP:66.1 ± 6.1	

Adverse Events

Only three of the four studies in this study reported adverse events [12,14-15]. No adverse events were recorded throughout the trials for RSE studies [12,14]. No adverse events were also recorded for the GSE supplement [15].

Discussion

This systematic review analyzed four studies, with a total of 94 patients and a mean follow-up of 43.8 days, to assess the outcomes of spinach extract supplementation on physical performance, body composition, and rates of complications. Previous studies on spinach extract investigated the acute effects of supplementation in short time frames, which may not represent its effects at longer-term follow-ups. The main findings in this study were that RSE (three studies) and GSE (one study) demonstrated significant improvements in physical performance parameters across most measured outcomes, mixed results on body composition, and no complications noted. These results suggest that spinach extract supplementation has practical ergogenic usability. However, these findings must be carefully interpreted as the number of included patients and follow-up time was small. To the best of our knowledge, this is the first systematic review examining the effects of spinach extract on exercise performance.

Physical Performance and Body Composition

The effects of exogenous NO3- supplementation on improving exercise performance are still debated in the literature. Dietary supplementation with NO3- or NO- precursors has become more common due to its purported effects on exercise performance [16]. Recent systematic reviews and meta-analyses have suggested an optimal dosage for performance benefits of 5 to 16.8 mmol (300 to 1041 mg), taken two to three hours before exercise. There also appears to be a dose-response relationship [8,17]. A recent systematic review found that NO3- supplementation improved explosive performance (lasting six seconds or less) in around 40% of sprint-based and resistance-based studies [6]. Sprint-based exercise showed greater improvements in short-term and longer-term studies, while resistance-based exercise studies only benefited from acute dosing. This difference may be due to variations in participants' training history, which lead to differences in motor unit recruitment patterns and muscle fiber type composition [18]. Recent literature supports this idea, suggesting that NO3- supplementation has the greatest benefit during the initial contraction, with more trained individuals experiencing greater speed of acceleration [6,19-20]. Differences in upper and lower body musculature may also impact the efficacy of NO3- [6].

Haynes et al. analyzed the effects of RSE on anaerobic bench press performance. They found no increase in muscle oxygenation and exercise performance but rather a subjective increase in focus and “muscle pumps” [12]. Linoby et al. investigated a 15-day course of 4 g RSE for aerobic exercise. They found that study participants had a significant increase in fractional exhaled NO- and time to exhaustion (TTE) with high-intensity exercise (p<0.001 and p=0.024, respectively) [21]. Similarly, Raymond et al. investigated the effect of 4.4 g of RSE on repeated high-intensity exercise performance in high-level collegiate soccer players [16]. It was found that although RSE did not enhance power output during repeated anaerobic exercise (sprinting), there were decreased fatigue indices (p=0.018) and increased post-exercise blood lactate levels (p=0.03), which may help in reducing physical fatigue. Increased blood lactate removal from muscle has been linked with monocarboxylate transporters, which are involved in the body’s adaptability to central fatigue [22]. These findings suggest that RSE may indirectly improve exercise performance by sustaining power output through decreasing fatigue indices.

In a study with the longest follow-up at 12 weeks, Perez-Pinero et al. found no significant changes in body composition overall; however, the male group did have a significant increase in muscle mass and isokinetic/isometric muscle strength compared to the female group when taking GSE [15]. GSE may offer an additional benefit as it provides a source of plant steroids, most notably ecdysterone (range: 17.1 to 885 ug/g dry weight), showing anabolic properties [23].

The study by Moore et al. involved using a single 1 g dose of RSE. During graded exercise training, it significantly increased plasma NO3- and ventilatory threshold (VT). However, no changes were observed in peak oxygen uptake or TTE [24]. Interestingly, there was no noticeable increase in plasma NO2- despite using high-sensitivity detection methods. Similarly, Martin et al. also found an increase in NO3- and VT with 1 g of RSE but no differences in peak oxygen uptake or TTE [25]. In contrast, a study by Linoby et al. found that 15-day supplementation with 4 g of RSE led to a 19% increase in TTE compared to the placebo group [21]. This suggests that RSE supplementation may improve sustained submaximal physical performance and potentiate higher training intensities. However, a study by Liubertas et al. found no significant changes in exercise capacity (cycling) despite increased peak power, VT, and maximal oxygen intake [26]. It is worth noting that previous studies have shown that peak plasma NO2- levels occur at around 2.5 hours post-ingestion. This indicates that the timing of supplementation of either NO3- or NO2- may be an important factor for future investigation [24].

Subramanian and Gupta's study showed that a single 2000 mg dose significantly increased plasma NO3- and NO2- levels to 253 umol/L and 0.56 umol/L, respectively, between 45 to 90 minutes [27]. The pharmacokinetic data from the same study suggested that NO3- and NO2- levels could start to increase as early as 30 minutes after ingestion and remain elevated for up to eight hours [27]. NO3- concentration was significantly higher in skeletal muscle than in plasma. Wylie et al. discovered that skeletal muscle NO3- levels decreased by 39% after high-intensity exercise, indicating its role in regulating NO3- levels during exercise [28]. This suggests that subacute and chronic NO3- ingestion could enhance this effect by increasing NO3- levels in skeletal muscle and potentially altering the expression/function of proteins regulating sarcoplasmic reticulum calcium release [29]. NO3- may also decrease the cost of phosphocreatine production and increase blood flow distribution to muscle fibers, thereby enhancing energy output efficiency and preserving power during high-intensity single and multiple repetitions [6]. Other potential mechanisms to improve oxygen delivery and utilization in skeletal muscles during exercise have been explored. Acute ingestion of 1000 mg of RSE increased lower limb microvascular reactivity one hour after ingestion, likely due to the increased usage of oxidative metabolism and subsequent increases in oxygen and blood flow requirements [30].

Increased NO- levels also improve muscular function by increasing vasodilation, blood flow, and oxygenation. Bentley et al. found that dietary NO3- led to compensatory vasodilation among patients with compromised oxygen delivery and improved perfusion and exercise capacity [31]. Furthermore, Pekas et al. showed that dietary NO3- improved endothelial function and walking capacity among patients with peripheral arterial disease [32]. The findings in a study with chronic obstructive pulmonary disease patients showed that dietary NO3- improved exercise capacity, most likely due to improved muscle oxygenation and vascular endothelial function [33].

Additionally, spinach's anti-oxidants, such as flavonoids, can also aid in improved recovery and muscle function by mitigating the oxidative stress induced by physical activity. A 2023 meta-analysis of Quercetin found that supplementation significantly decreased muscle soreness (standardized mean difference: -1.33, p=-0.03), creatine kinase levels (standardized mean difference: -1.15, p=0.02), and post-exercise oxidative stress (standardized mean difference: -0.92, p=0.03) [34]. These findings are supported by a 2021 meta-analysis of flavonoid recovery potential, which found improved muscle strength recovery by 7.14% and reduced muscle soreness by 4.12% (p<0.001 and p=0.001, respectively) [35]. Thus, the ergogenic benefits of spinach may be attributed to multiple components, but more human studies are required to confirm this.

Most current inorganic NO3- studies have used beetroot juice [16,21]. There is widespread evidence that acute beetroot juice supplementation improves exercise performance, including increased velocity, power, repetition volume, agility, sprint performance, and anaerobic capacity [36-38]. However, a concentrated supplement dosage must be ingested to illicit the reported effects. Thus, there is a possibility that marketed products online may not contain a clinical therapeutic level of NO2- or NO3- to produce a noticeable effect [37]. Beetroot is not the only high-quality source of NO3- available. As covered in this study, RSE and GSE are promising alternatives.

Research suggests that RSE may contain similar or even higher NO3- levels than beetroot [39]. A study from 2016 supports this claim by showing a 255% increase in blood NO3- levels with RSE, which is said to have up to four times more NO3- than beetroot [30]. Unlike beetroot juice, RSE is rich in minerals such as potassium and magnesium and does not contain sugar or oxalates. The high potassium content has been suggested to contribute to exercise-induced hyperemia [36]. RSE also contains unique polyphenols like amaranthine and different relative levels of compounds such as quercetin compared to other sources of NO3- [24]. The potential effects of RSE on exercise performance and pharmacodynamics are not yet fully understood and require further investigation.

Adverse Events

The benefits of NO3- have been well documented; however, their supplementation is subject to upper limits. The acceptable daily intake for NO3- and NO2- is 3.7 mg/kg of body weight and 0.07 mg/kg of body weight, respectively. Excessive NO3- can lead to methemoglobinemia and the formation of carcinogenic nitrosamines if combined with amines [23]. Beetroot, RSE, and GSE contain varying levels of NO3-: 2500 mg/kg, 2800 to 8800 mg/kg (in leaves), and 900 to 5400 mg/kg (in leaves) [23]. NO3- supplementation has been found to lower blood pressure after exercise in active and resting normotensive adults [14]. Despite the high levels of NO3- in RSE, there is speculation that it may not lead to significant alterations in blood pressure [36,40].

Implications

Using bioactive ingredients in food or supplements for ergogenic purposes has garnered substantial interest among recreational and professional athletes, aiming to enhance exercise performance and expedite recovery. Research indicates dietary NO3- has improved physical performance, even among non-athletes [8]. However, Porcelli et al. observed a notable enhancement in aerobic fitness among individuals with low to moderate levels of training, while highly trained individuals did not demonstrate significant benefits [41]. This discrepancy may be attributed, at least in part, to the more optimal expression and activity of NO- in highly trained populations [9].

The following considerations should be taken into account in future studies. Firstly, the dietary habits of patient cohorts before the study should be carefully considered, particularly concerning variable vegetable consumption and low levels of baseline dietary intake of NO3-, which may impact the effectiveness of NO3- supplementation. Co-ingestion with other ergogenic substances could also affect NO3- bioavailability, although limited data is currently available, necessitating further research. Additionally, factors such as dose regimen (acute/subacute/chronic), vehicle of administration (juice versus salts), and bioactive substances like polyphenols should be examined for their potential influence. Furthermore, the impact of training status on baseline plasma NO3- and bioavailability should be acknowledged. To compare their outcomes, future studies should involve varying levels of exercise intensity protocols between different sources of NO3-, such as RSE, GSE, and beetroot. Moreover, alternative or potentially superior sources should be considered. Dosing regimens, various exercise modalities, and gender differences should also be explored in these studies [6].

Limitations

The findings of this study should be interpreted within the confines of its limitations. First, the study had a small sample size of athletes, which may not represent the general population. The follow-up period was also relatively short, preventing the determination of its long-term effects, sustainability, and safety profile over time. Therefore, larger studies with extended follow-up periods are necessary to generalize these findings and comprehend the long-term effects of supplementation. Second, variations in baseline patient demographics and NO- levels may exist based on age, gender, diet, and athletic background. Consequently, making definitive statements about the effectiveness of spinach extract is challenging. The NO3- content and composition of each extract in the included studies also significantly varied. Further studies should standardize the intervention protocols and measurement outcomes to facilitate comparisons and improve the findings' generalizability. Third, due to heterogeneity in the PROs, a meta-analysis could not be conducted.

Furthermore, non-English studies were not included, and there is a possibility of unpublished completed trials not encompassed in this review. Fourth, when assessing the RoB, inadequate random sequence generation and allocation concealment can influence baseline characteristics between groups and group assignments, affecting the treatment effects seen. Additionally, although the studies were stated as double-blinded in design, the exact description was not detailed, which can bias behaviors and outcome measurements. Thus, these findings may affect the spinach extract’s effectiveness and provide an additional challenge when interpreting the true magnitude of the treatment effects.

## Conclusions

Spinach extract exhibits promising physical performance advancements and is a safe ergogenic aid. These benefits are primarily ascribed to enhanced NO3- bioavailability, which age, dietary patterns, and athletic history can influence. To enhance the applicability and comprehension of these findings, forthcoming research should prioritize standardizing baseline patient demographics and investigating the impact of dosage regimens, types of physical activity, and gender differentials. Further investigation is necessary to ascertain the optimal dosage schedules for spinach extract and its impact on short-term and long-term physical performance.
